# Optic Neuritis Following Nivolumab Treatment for Metastatic Melanoma: A Case Report and Review of the Literature

**DOI:** 10.7759/cureus.92212

**Published:** 2025-09-13

**Authors:** Themis Gwendolyne Aguilar-Arciga, Gerardo Chavira-Hernández, Victor Antonio Mendoza-Vargas, David Ancona-Lezama

**Affiliations:** 1 Ophthalmology Department, Hospital Regional “Dr. Valentín Gómez Farías”, Instituto de Seguridad y Servicios Sociales de los Trabajadores del Estado (ISSSTE), Zapopan, MEX; 2 Neurology Department, Hospital Regional “Dr. Valentín Gómez Farías”, Instituto de Seguridad y Servicios Sociales de los Trabajadores del Estado (ISSSTE), Zapopan, MEX; 3 Internal Medicine Department, Hospital Regional “Dr. Valentín Gómez Farías”, Instituto de Seguridad y Servicios Sociales de los Trabajadores del Estado (ISSSTE), Zapopan, MEX; 4 Ophthalmology Department, Eye Cancer Institute, San Pedro Garza García, MEX

**Keywords:** immune checkpoint inhibitors (icis), metastatic melanoma, nivolumab, non-infectious uveitis, optic neuritis

## Abstract

Immune checkpoint inhibitors have revolutionized the treatment of melanoma and other tumors; however, these novel drugs can trigger immune-related adverse events (irAEs), involving uncontrolled autoimmune cross-reactive damage to the brain, peripheral nerves, eyes, and connective tissue.

We report an unusual case of optic neuritis and anterior uveitis following treatment with nivolumab, an anti-programmed cell death protein 1 inhibitor, in a 60-year-old Mexican woman with metastatic melanoma. We ruled out infectious, autoimmune, inflammatory, and neoplastic etiologies as the cause of optic neuritis. The patient experienced significant improvement in visual acuity and ocular pain after discontinuing nivolumab and initiating steroids with a tapered dosing regimen.

When using checkpoint inhibitors, ocular and neurological symptoms should be monitored, and corticosteroid therapy can be considered a first-line treatment for patients with irAEs associated with nivolumab.

## Introduction

T cells express surface molecules known as checkpoints, which can prevent cell death during immune surveillance. Melanoma is a malignancy of melanocytes that can evade immune surveillance by activating checkpoints on T cells. Immune checkpoint inhibitors (ICIs) are novel drugs that block the activation of these checkpoints, allowing the immune system to destroy tumor cells [1]. Checkpoint inhibitors include monoclonal antibodies that block and inhibit cytotoxic T-lymphocyte-associated antigen 4 (ipilimumab, tremelimumab) or anti-programmed cell death protein 1 (anti-PD-1) inhibitors (nivolumab, pembrolizumab) [2].

Tumors treated with ICIs include melanoma, small cell lung cancer, metastatic colorectal cancer, esophageal adenocarcinoma, esophageal squamous cell carcinoma, gastric cancer, head and neck squamous cell carcinoma, hepatocellular carcinoma, Hodgkin lymphoma, pleural mesothelioma, renal cell carcinoma, and urothelial carcinoma. However, ICI antibodies can cause excessive and uncontrolled cross-reactive immune responses, known as immune-related adverse events (irAEs). irAEs are mediated by overactivation of T cells due to the loss of checkpoint-mediated inhibition in non-cancerous cells. They are estimated to occur in fewer than 3% of patients [3], with ocular and nervous system irAEs being even rarer, occurring in approximately 1% of patients treated with ICIs [4].

Uveitis, orbital myositis, Vogt-Koyanagi-Harada disease, myasthenia gravis, optic neuritis, chronic inflammatory demyelinating polyneuropathy, transverse myelitis, optic atrophy, exudative vitelliform maculopathy, and choroidal neovascularization are among the most frequently described neurologic and ocular irAEs [5]. These neurological and ocular comorbidities can leave irreversible sequelae such as weakness, scotomas, decreased visual acuity, double vision, and blindness. Therefore, their identification and treatment are of vital importance in the clinical setting.

Herein, we report the case of a female patient with metastatic melanoma treated with nivolumab who developed optic neuritis and anterior uveitis.

## Case presentation

We present the case of a 60-year-old Mexican woman with a history of tobacco use and no family history of neurological disorders. In June 2022, she was diagnosed with metastatic nodular cutaneous melanoma on her right dorsal back (T3a, N1, M1, stage IIIB; tumor thickness 2.7 mm; Clark level IV). Two months later, a metastatic lesion was detected on her right arm and surgically removed. The patient began treatment with 480 mg of intravenous nivolumab every four weeks. Three weeks after the seventh monthly dose of nivolumab, she presented to the emergency department with blurred vision, decreased visual acuity, and orbital pain associated with eye movements that had developed over three days.

**Figure 1:**
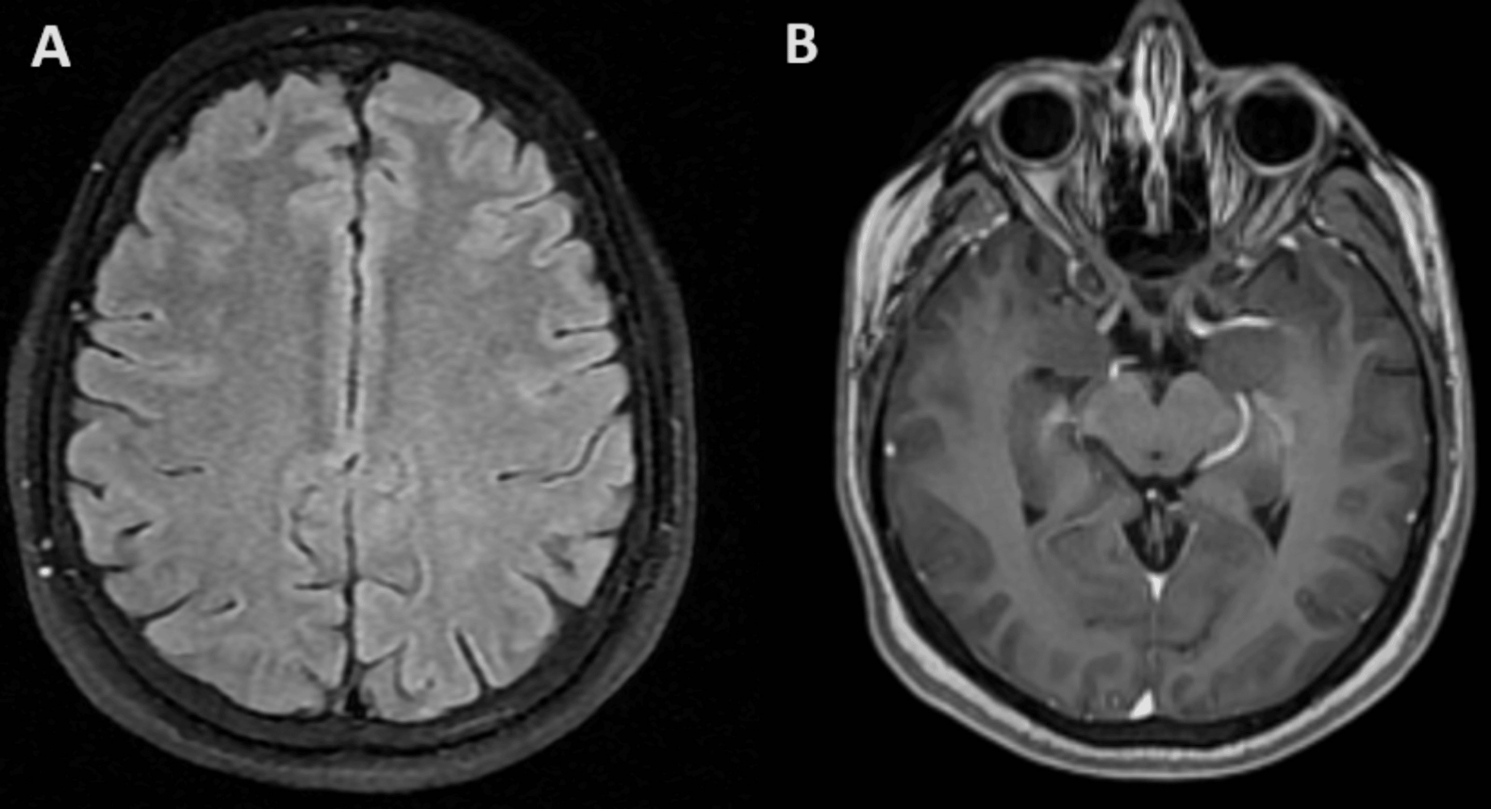
Magnetic resonance imaging (A) Fluid-attenuated inversion recovery (FLAIR) cortical sequence showing no evidence of demyelinating lesions in the cortical-subcortical regions of the frontoparietal lobes. (B) Contrast-enhanced T1 sequence showing no evidence of optic nerve or chiasm enhancement.

On examination, her visual acuity was 20/80 in the right eye and 20/150 in the left eye, with a bilateral relative afferent pupillary defect. Pharmacologic examination of the pupils revealed papilledema without vitritis. Intraocular pressure was 12 mmHg in the right eye and 14 mmHg in the left eye. The remainder of the neurological examination was normal. Humphrey visual field testing revealed an enlarged blind spot and decreased peripheral sensitivity in both eyes. Several investigations were performed to rule out other causes of optic neuritis. Complete blood count was normal, and a rheumatologic panel was negative for autoantibodies. Gadolinium-enhanced magnetic resonance imaging (MRI) of the brain and optic nerves showed no demyelinating lesions or optic nerve enhancement (Figure 1). Lumbar puncture revealed normal opening pressure; cerebrospinal fluid (CSF) total cell count was 1; cultures were negative; cytologic evaluation showed no malignant cells; and polymerase chain reaction testing was negative for viruses, bacteria, and fungi. CSF oligoclonal bands, serum anti-aquaporin-4 (AQP4) antibodies, and serum anti-myelin oligodendrocyte glycoprotein (MOG) antibodies were all negative. Whole-body positron emission tomography (PET) showed no evidence of macroscopic melanoma activity, neither ocular nor meningeal infiltration.

Optic neuritis secondary to ICIs was diagnosed after excluding infectious, autoimmune, inflammatory, and neoplastic causes. Non-treponemal (venereal disease research laboratory, or VDRL) and treponemal (fluorescent treponemal antibody absorption, or FTA-ABS) serologic tests were negative. CSF analysis revealed normal adenosine deaminase levels, and mycobacterial culture yielded no growth. Accordingly, syphilis and tuberculosis - frequent mimickers of infectious optic neuropathy - were excluded. Nivolumab treatment was permanently discontinued, and the patient received intravenous methylprednisolone 1 g daily for five consecutive days. Ocular pain resolved, and visual acuity improved to 20/40 in the right eye and 20/30 in the left eye.

**Figure 2:**
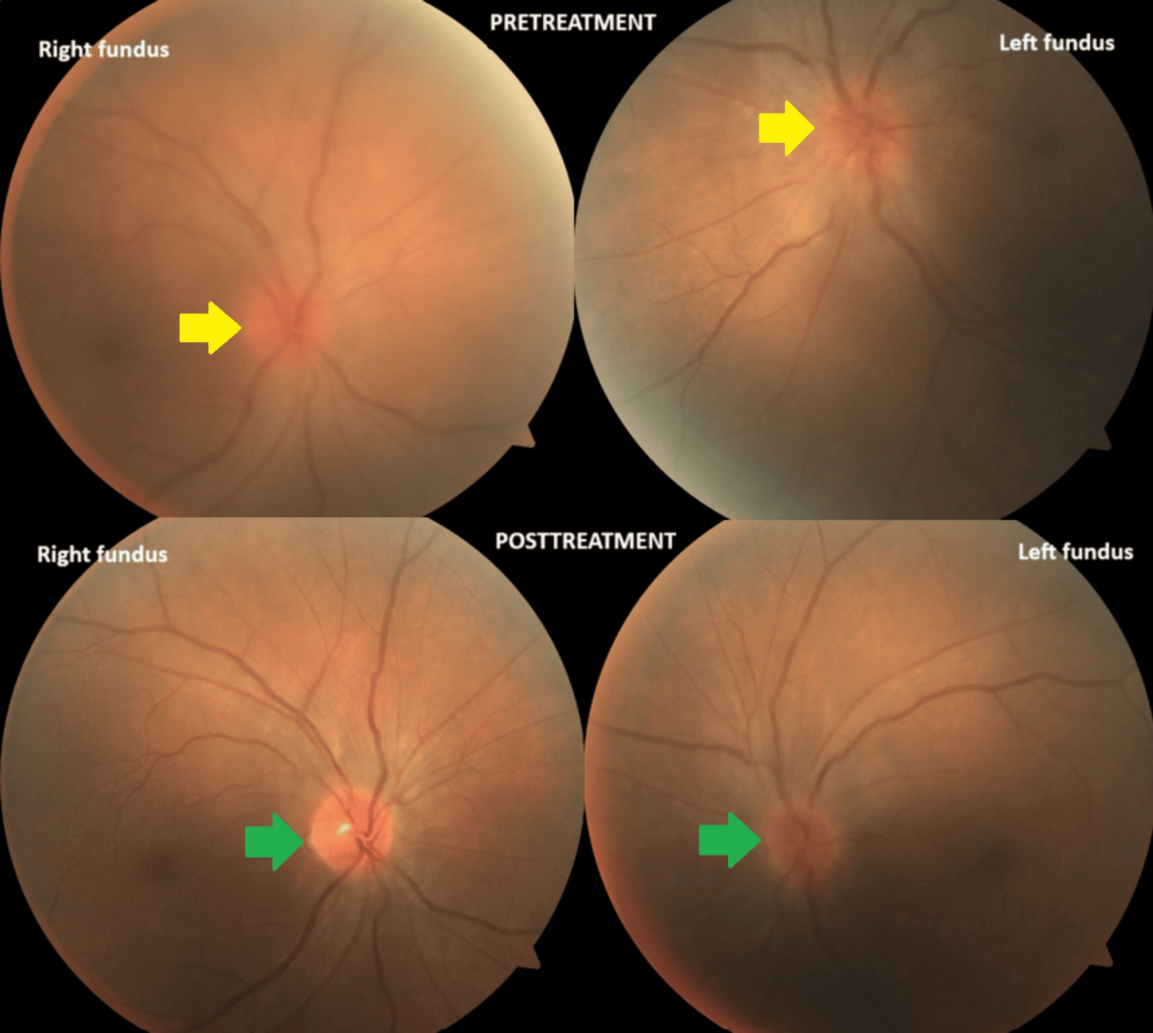
Images of the patient’s eye fundus before and after treatment Fundus images of both eyes show papillitis with blurred borders and loss of the neuroretinal rim (yellow arrows). After therapy, there is improvement in the optic disc borders (green arrows), but also the presence of an atrophic, pale-yellow optic nerve, particularly in the right eye.

**Figure 3:**
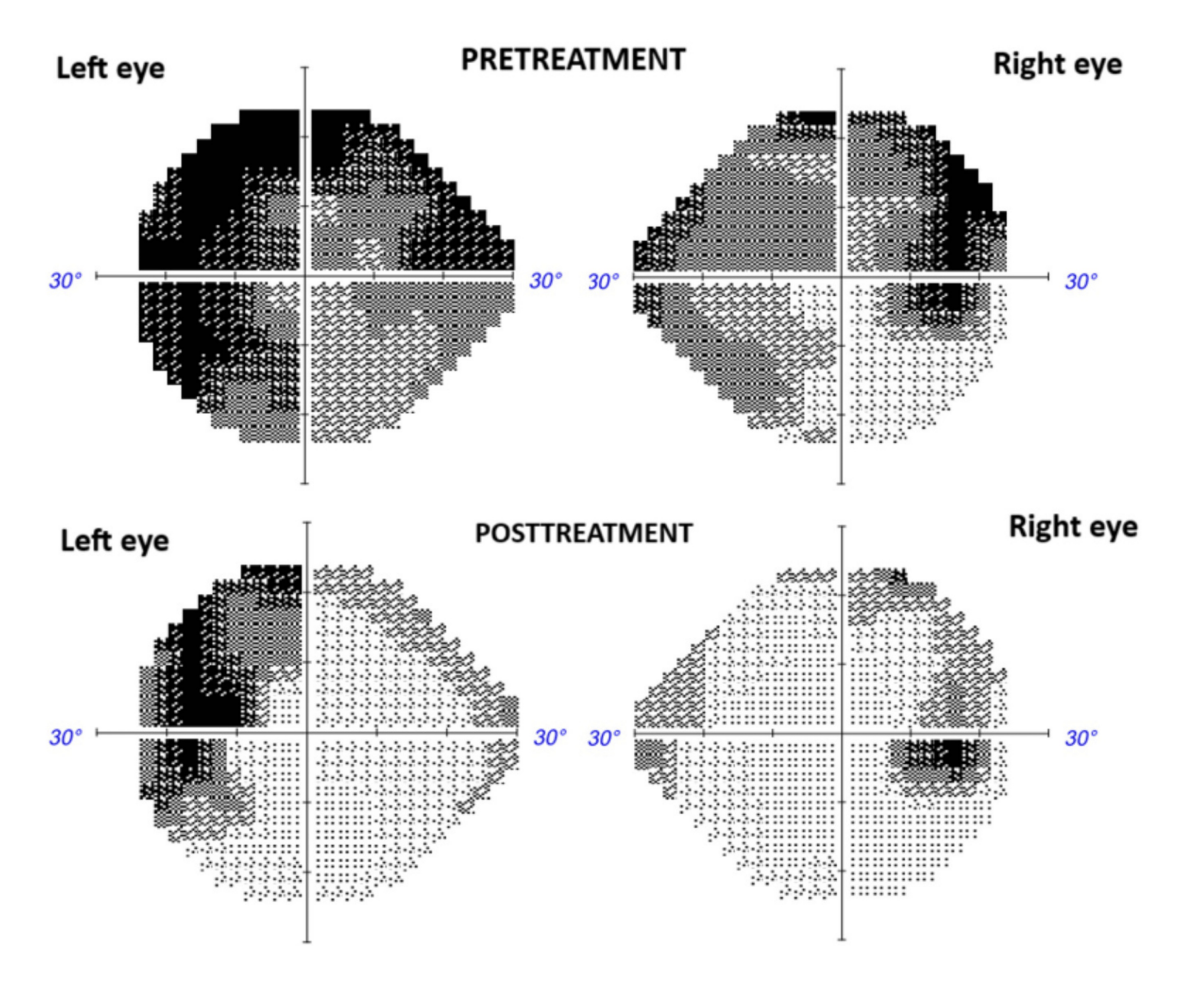
Images of the patient’s visual fields before and after treatment Humphrey Visual Field testing revealed an enlarged blind spot and decreased peripheral sensitivity in both eyes. Both the visual fields and the blind spot improved after steroid therapy.

After discharge, the patient was started on oral prednisone 1 mg/kg daily in a four-week tapering regimen, with further improvement in visual acuity to 20/20 bilaterally. Optic disc edema (Figure 2) and scotomas (Figure 3) also improved in both eyes.

One month after discontinuing oral prednisone, the patient presented to the ophthalmology department with a mild episode of non-granulomatous anterior uveitis in the left eye, which was treated with cycloplegic agents and topical corticosteroids, leading to complete resolution after two weeks.

## Discussion

Ocular and neurologic irAEs are uncommon complications of ICIs, yet their potential to cause irreversible sequelae warrants prompt recognition and intervention. These adverse effects have been reported in approximately 1% of patients, with an average onset of six weeks after the first dose; however, onset can range from as early as one week to as late as five years after treatment initiation [6].

The European Society for Medical Oncology and the American Society of Clinical Oncology recommend discontinuing ICIs and initiating high-dose intravenous steroids. Steroids reversibly inhibit inosine monophosphate dehydrogenase, an enzyme involved in guanosine synthesis, thereby reducing T- and B-lymphocyte proliferation and maturation [7,8].

MRI findings in patients with irAEs can vary; some may demonstrate subependymal, optic nerve, or meningeal enhancement, while others present without imaging abnormalities [9-12]. In many cases, including ours, early discontinuation of ICIs and initiation of high-dose corticosteroids have been associated with significant visual and neurological recovery (Table 1).

**Table 1:** Previously reported cases of optic neuritis secondary to nivolumab AQP4: anti-aquaporin-4; IV: intravenous

Author	Age	Comorbidities	Clinical features	Indication	Nivolumab dose	MRI	Treatment	Response
Nowosielski et al. [9]	Male, 47 years	None	Arthralgia, facial peripheral paralysis, blurry vision	Melanoma	Nivolumab 3 mg/kg (between July 2018 and February 2019) (13 cycles); ipilimumab 3 mg/kg every three weeks since March 2019 (three doses)	Enhancement of cranial nerve roots C2/C3 and cauda equina. Subependymal and nodular parenchymal contrast-enhancing lesions	Methylprednisolone for one month, rituximab 1000 mg (two doses)	Remission
Kartal and Ataş [10]	Male, 9 years	None	Visual impairment in both eyes, bilateral disc swelling	Glioblastoma multiforme (as third-line treatment)	Nivolumab 2 mg/kg (two cycles)	Bilateral thickening of the optic nerves	Methylprednisolone 1 g IV (five days)	Improved symptoms
Ahluwalia and Kohli [11]	Female, 35 years	None	Photopsia in the right eye, papilledema in the right eye	Melanoma	Nivolumab (eight cycles - no dosage specification)	Normal (without enhancement of optical nerves)	Methylprednisolone 1 g IV (three days), oral prednisone (tapered)	Improved symptoms
Narumi et al. [12]	Male, 75 years	None	Acute paralysis of lower limbs, sensory loss below T10, and urinary retention. Neuromyelitis optica spectrum disorder (positive serum AQP4 immunoglobulin G antibodies)	Lung carcinoma (as second-line treatment)	Nivolumab (3 mg/kg) for one dose	Normal (without enhancement of optical nerves)	Steroid pulse therapy (no specified dosage), plasmapheresis	Improved symptoms
Francis et al. [4]	Female, 61 years	Not reported	Decreased vision, “smudge”	Cutaneous melanoma	Nivolumab 1 mg/kg for three weeks	Not reported	Not reported	Unknown
	Female, 58 years	Not reported	Decreased vision, halos	Cutaneous melanoma	Nivolumab 1 mg/kg for three weeks	Not reported	Not reported	Unknown
	Female, 59 years	Not reported	Decreased vision, “large" floater	Small-cell lung carcinoma	Nivolumab 240 mg for three weeks	Not reported	Not reported	Unknown
	Female, 54 years	Not reported	Decreased vision	Small-cell lung carcinoma	Nivolumab 240 mg for three weeks	Not reported	Not reported	Unknown
	Male, 68 years	Not reported	Peripheral vision loss	Cutaneous melanoma	Nivolumab 1 mg/kg for three weeks	Not reported	Not reported	Unknown
	Male, 65 years	Not reported	Decreased vision	Cutaneous melanoma	Nivolumab 1 mg/kg for three weeks	Not reported	Not reported	Unknown
	Male, 63 years	Not reported	Decreased vision, redness	Cutaneous melanoma	Nivolumab 2 mg/kg for two weeks	Not reported	Not reported	Unknown
	Male, 58 years	Not reported	Decreased vision	Small-cell lung carcinoma	Nivolumab 1 mg/kg for three weeks	Not reported	Not reported	Unknown

## Conclusions

Although ocular and neurologic irAEs remain rare, they can be disabling or even life-threatening. Prompt recognition, exclusion of infectious, autoimmune, and neoplastic mimics, and rapid initiation of therapy are critical to preventing long-term disability. Clinicians should be aware of the potential causes of optic neuritis and ensure they are excluded before confirming the diagnosis of irAEs. In this case, we emphasize the importance of maintaining a high index of suspicion for irAEs in patients receiving nivolumab, regardless of the time elapsed since therapy initiation. Early discontinuation of nivolumab and timely initiation of steroids or other immunomodulatory therapies can lead to functional recovery, as illustrated by our patient.

Multidisciplinary collaboration between oncologists, neurologists, and ophthalmologists is essential for early detection, accurate diagnosis, and optimal management, thereby minimizing the risk of permanent sequelae. It is important to mention that this is a single case report. While it highlights a clinically significant irAE, the findings should not be overgeneralized. Major limitations of this report include the lack of long-term follow-up with a high risk of recurrence, progression of optic atrophy, and incomplete exclusion of genetic causes. These are in addition to the inherent limitations of case reports, such as subjective interpretation and limited reproducibility. More robust evidence from randomized controlled trials and meta-analyses is needed to guide therapeutic management in these patients.
